# A longitudinal study of the implementation experiences of the Australian National Disability Insurance Scheme: investigating transformative policy change

**DOI:** 10.1186/s12913-017-2522-7

**Published:** 2017-08-17

**Authors:** Gemma Carey, Helen Dickinson

**Affiliations:** Public Service Research Group, University of New South Wales: Canberra, Nothcott Drive, Canberra, 2600 Australia

## Abstract

**Background:**

Internationally there has been a growth in the use of publicly funded service markets as a mechanism to deliver health and social services. This has accompanied the emergence of ‘self-directed care’ in a number of different policy areas including disability and aged care – often referred to as ‘personalisation’ (Giaimo and Manow, Comp. Pol Stud 32:967–1000, 1999; Needham, Public Money Manage 30:136–8, 2010; [Hood], [The Idea of Joined-up Government: A Historical Perspective], [2005]; Klijn and Koppenjan, Public Manage 2:437–54, 2000, Greener, Policy Polit 36:93–108, 2008). These reforms are underpinned by an idea that individuals should be placed in control of their own service needs, given funding directly by government and encouraged to exercise choice and control through purchasing their own services. A major challenge for governments in charge of these reforms is determining the best way to structure and govern emerging service markets markets. Given the growing international embrace of market-based reform mechanisms to provide essential services to citizens, finding ways to ensure they promote, and not diminish, people’s health and wellbeing is vital.

**Methods:**

The Australian National Disability Insurance Scheme (NDIS) is Australia’s first national approach to the use of personalised budgets. The program of research outlined in this paper brings together streams from a range of different studies in order to investigate the implementation of the NDIS longitudinally across different administrative levels of government, service providers and scheme participants.

**Conclusion:**

This programme of research will make a contribution to our understanding of the Australian scheme and how individualised funding operates within this context, but will also generate much needed evidence that will have relevance to other jurisdictions and help fill a gap in the evidence base.

## Background

In many countries around the world, welfare states – the mechanism through which governments protect and provide for their citizens – are in a state of critical transition. Faced with a range of fiscal and social pressures, industrialised countries are moving away from the collective social provision that drove the development of post-war welfare states [1] and which functioned as a social safety net, providing a range of services and financial assistance directly to citizens [2].

In response to these various pressures we have seen the increasing use of markets as a mechanism to deliver welfare services and the emergence of ‘self-directed care’ in a number of different policy areas – often referred to as ‘personalisation’ [3–7]. These reforms are underpinned by an idea that individuals should be placed in control of their own service needs, given funding directly by government and encouraged to exercise choice and control through purchasing their own services. Self-directed care has become central to public service delivery in a wide range of countries and policy areas, from the National Health Service in the England to the *Brukerstyrt Personlig Assistanse* in Norway [8–11].

Where personalisation reforms are in place citizens are given money directly by government and must negotiate their own use of services; sourcing, understanding and choosing services that best meet their needs from a range of private and not-for-profit providers. Although these schemes have been in place in different jurisdictions for some time now, our knowledge of the implications of this shift is still in its infancy, both for citizens negotiating these new markets and for the governments overseeing them [8, 12]. A recent systematic review of personal budgets for disability care found that most empirical research did not include detail about funding mechanisms, and that it is difficult to distinguish between processes and outcomes in some studies [13]. Differential outcomes have already been shown to emerge from personalisation approaches in the UK according to the capabilities and existing supports of the individual [4].

One of the challenges inherent in these reform processes is determining the best way to structure and govern such approaches. Governments are often required to balance control and flexibility to ensure markets function effectively and equitably. Therefore market-based welfare reforms, and the systems that govern them, are required to find ways to change, learn and adapt as these contexts evolve [14]. To do this, governments’ seek market ‘levers’ that they aim to adjust in order to correct market failures [15]. However, as economist Joseph Stiglitz has argued, governments find markets notoriously difficult to regulate and manage in predictable and reliable ways [16]. This makes the use of markets within a social services context risky. Given the growing international embrace of market-based reform mechanisms to provide essential services to citizens, finding ways to ensure they promote, and not diminish, people’s health and wellbeing is vital.

The Australian National Disability Insurance Scheme (NDIS) is Australia’s first national approach to the use of personalised budgets in this policy area (Dickinson & Needham, Forthcoming). Consistent with changes in the UK and Europe, the NDIS represents a transition to self-directed/personalised care: over 460,000 individuals with mental and physical disabilities will have to navigate a newly created service market in order to gain the assistance they need [17]. Launched in 2013, the NDIS represents a rare opportunity to study current large-scale transitions in social welfare provision. The Australian experience is unprecedented in several important ways. Firstly, the geographical spread outstrips that of other countries. Secondly, the Australian scheme is combined in an insurance approach [18]. Hence the scale of the NDIS is broader and deeper than its international counterparts running at a cost of over $22 billion a year, offering important opportunities for learning.

## Study design

This on-going program of research reported in this paper brings together streams led by the authors from a range of different studies in order to investigate the implementation of the NDIS longitudinally for a period of 5^1^ years across different administrative levels of government, service providers and scheme participants.^2^

The project has three **objectives**:

1. To investigate the implementation experiences of key actors (e.g. Federal and State policymakers, NDIS administrators and service providers) with regard to NDIS governance structures, paying particular attention to the responsiveness and adaptation of such structures;
2. Explore the experiences of stakeholders (including scheme participants) involved in the establishment of new public sector disability markets with a view to examining:

- Determinants of success and failure for care service providers and care outcomes
- pathways and processes for effective market management (i.e. addressing thin markets and market failure)

## Understand the opportunities and/or limits of markets for the provision of public services and, in turn, the future structure of the welfare state

### Context and theoretical framework

This project is embedded within the field of public policy and administration and draws on theories of new public governance (NPG) [19] to frame the broad study. The strength of NPG is its basis in network and institutional theoretical perspectives assists in capturing the real world complexity of the design, implementation and management of public policy in the twenty-first Century [19–21]. In this sense, NPG encapsulates both emerging public policy implementation and public service delivery challenges *and* issues left unresolved within previous iterations of public sector governance (including 1970/1980s public administration reforms and 1990s ‘new public management’ approaches) [19, 22].

NPG conceptualises a *plural* state that has multiple interdependent actors contributing to the delivery of services, as well as a *pluralist* state with multiple processes inform the policy-making system [19]. These two types of plurality mean the focus within an NPG approach is on inter-organisational relationships and governance processes. Indeed, since the 1970s organisational analysis has thought to be critical to understanding why particular outcomes emerge from reform efforts as a result of implementation processes [23, 24]. However, within an NPG framework both the actors and their relational processes are situated within institutional and environmental contexts that work to enable and constrain policy implementation, shaping the negotiation of trust, values and meaning within and between organisations [19, 22]. The value of this approach within the context of this study is that it allows us to accommodate consideration of both formalised structures within the system and agency of the various actors involved in these reform processes.

### Data collection

#### Scope

The study will examine the implementation of the NDIS from a range of vantage points, capturing the emergent dynamics between institutional contexts and implementation processes [22]. An iterative approach to data collection will is taken in order to capture change over time. Our in-depth and multi-site approach enables the research team to track reform dynamics through an analysis of the type of tacit knowledge that is rarely captured during implementation (or time limited evaluations) but which ultimately shapes the trajectory of both current and future reforms [25, 26].

The study has six interrelated components that seek to capture implementation and user experiences across the different domains of the NDIS

### Document review

A review of documents relating to the implementation and evaluation of the NDIS will be undertaken throughout the project, to identify how emerging knowledge and practices are responded to and alter implementation and governance/market architectures. This analysis will also reveal how the NDIS is understood to exist both as a social program in its own right, and in relation to other programs and policies, and whether this changes over time. Documents will include: all available documents sourced through the NDIA (i.e. evaluation reports, strategic plans, program reports and funding agreements), in addition to publicly available evaluation and implementation reports. These will be collected and analysed in conjunction with other data sources throughout the duration of the project.

### Semi-structured qualitative interviews with commonwealth and state government officials

Interviews with individuals embedded in Commonwealth and State agencies are key to understanding how the introduction of the NDIS is reshaping the broader social protection framework, and the implications of this reshaping for both fairness and the implementation of future reforms. Interviews with key actors will identify the effect of the NDIS on national and State priorities, funding decisions and program development. Interviews will also enable the research team to understand the operation of the governance structures, and the challenges associated with balancing flexibility, control and accountability.

Criterion-based, purposive sampling [26] of individuals will be conducted chosen on the basis of current/past role in State and Commonwealth administration (e.g. Departments of Human Services and central coordinating agencies such as the Department of Prime Minister and Cabinet).

Snowball sampling will be carried out as participants will be asked to nominate other stakeholders, until saturation is reached [26]. Final sample size will be determined by saturation. Interviews will be individual and semi-structured. Participants will be invited to be re-interviewed each 8 months (and further participants as appropriate) over 5 years in order to capture new information, knowledge and practices as implementation continues (minimum *N* = 100).

### Network analysis and interviews with service providers

Interviews with disability service providers in the three sites (Australian Capital Territory, Victoria and Queensland) will provide information on

- How care service organisations are responding and adapting to the new market context, and the implications for care outcomes
- Where and why thin markets or market failure emerges

The comparative approach will enable the research to investigate how governance and market architectures are able to manage different types of market variation or service issues. Each of the three sites have location-specific governance and funding arrangements.

Up to 20 service providers will be interviewed in each site 1 a year, accompanied by a network analysis survey (consistent with network analysis sampling techniques) [27]. Network analysis provides a tool for measuring and analyzing network structure, changes and potential effectiveness [27, 28]. Network analysis has two components. Firstly, a structured component to generate network data – questions are directed at identifying network structure and function in the disability sector. Secondly, a semi-structured component with open-ended responses to questions about the experience of implementation for service providers and the new network structure. This will enable the research to identify how and why network structures are changing and identifying emerging forms of collaboration between services. The open-ended questions will seek to identify the barriers and facilitators to innovation. Moreover, they will determine whether emerging governance structures are indeed collaborative (versus a contractual or hierarchical arrangements) and if they support true innovation.

The social network analysis survey will be distributed online once a year in all three sites, using registered provider lists available through the National Disability Insurance Agency.

### Peer-research with scheme participants

In order to capture the experiences of scheme participants, community researchers will be used to gain a deep appreciation of the impact of NDIS reforms to disability services from the perspective of consumers of these services and their families.

Participatory research processes are used, involving community researchers who do not know research participants but who had a common experience of accessing disability services. Such an approach was embraced on the basis that it should help increase the likelihood of uncovering issues and challenges faced by service users [29, 30]. The aim is to encourage interactions with research participants that are respectful, supportive and interactive conversations between peers, to elicit information about service user experiences of the NDIS using semi-structured interviews within one of the trial areas, sample determined by feasibility (*N* = 42).

The service users interviewed for the project fall into three categories: people with physical disabilities; people with intellectual disabilities or mental health issues; and, carers. Findings from this project will be fed back to a number of government agencies and will also provide the basis of a learning lab that seeks to explore the meaning of choice within individualised funding systems with a range of partners from across the disability system.

### Delphi analysis of stakeholders

An interactive Delphi approach will be used as a research translation method. Within a Delphi approach multiple iterations of data-collection and engagement are undertaken with a range of stakeholders. Delphi study designs overcomes the problem of single viewpoint and problem framing [31], enables new, effective and acceptable solutions to emerge from and in conjunction with complex stakeholder networks. The iterative process of conducting the Delphi study creates the opportunity for stakeholders to hear the views and ideas of others from very different sectors and settings and gain information on policy silences. This generates opportunities for new ideas and understanding, as well moving stakeholders towards a consensus.

Approximately 40 individuals will be included in the Delphi study (sample number determined by willing participants), drawn from government and non-government organisations, statutory bodies, disability service sector, Disabled People’s Organisations (advocacy groups). Stakeholders will be engaged twice a year in face-to-face interviews, and surveyed electronically in between. This iterative design will enable the research team to keep abreast of the rapidly developing policy environment, occurring as a result of the launch of the NDIS. This will include planned changes in disability policy, monitoring and data collection procedures, and potential areas of contestation that the research team may assist in shedding light on. Snowball sampling will be conducted (where participants nominate other participants), beginning with members of participants from other elements of the study – then engaging more broadly over the course of the program of research.

One-on-one interviews will enable us to capture critical policy silences, while exploring potential solutions emerging from other aspects of the research. In this sense, the Delphi study will support research translations and enabling innovative solutions to come to the fore.

### Analysis

All interviews and focus groups will be recorded and transcribed verbatim [26]. The aim is to uncover the tacit, or mutual, knowledge, intentions and rules that emerge from formal institutions and form the basis for emergent informal institutions – thereby shaping implementation action [26, 32]. Analysis will be guided by a range of theoretical frameworks which seek to elucidate how tacit knowledge shapes action, including new institutionalism, structuration theory and diffusion of innovation theory [22, 33, 34]. Data collected through the core research activities will be analysed iteratively. For example, documents and surveys will be reviewed in accordance with the themes identified and developed through the interview data.

### Ethics, consent and permissions

The research has ethics approval from the University of New South Wales Human Ethics Committee (No.: HC16396). All participants are required to sign a consent form prior to interviews agreeing to recording and use of the data in publications and conference presentations. Survey participants consent to participation through an online checkbox.

## Discussion/contribution

How to ensure the gains promised by major policy reforms are achieved and benefit the population is a contested and important area of inquiry. By investigating, and contributing to, how implementation can be secured this study contributes our knowledge of policy implementation, particularly in the context of major reforms**.** The new reform context that emerges from its implementation will govern not only what policies are adopted into the future, but also the likelihood that they will be able to secure gains for the community [22]. In other words, understanding the institutional shifts that occur as a result of the NDIS is essential to the successful implementation of all social welfare and social service reforms that come after it. This programme of research will make a contribution to our understanding of the Australian scheme and how individualised funding operates within this context, but will also generate much needed evidence that will have relevance to other jurisdictions and help fill a gap in the evidence base.

## Abbreviations

NDIS: National disability insurance scheme

## Footnotes

1 Extension of the study is contingent upon funding.

2 See Funding section below for list of studies.
